# Novel Efficient Bioprocessing of Marine Chitins into Active Anticancer Prodigiosin

**DOI:** 10.3390/md18010015

**Published:** 2019-12-23

**Authors:** Van Bon Nguyen, Shan-Ping Chen, Thi Hanh Nguyen, Minh Trung Nguyen, Thi Thanh Thao Tran, Chien Thang Doan, Thi Ngoc Tran, Anh Dzung Nguyen, Yao-Haur Kuo, San-Lang Wang

**Affiliations:** 1 Division of Computational Mathematics and Engineering, Institute for Computational Science, Ton Duc Thang University, Ho Chi Minh City 700000, Vietnam; nguyenvanbon@tdtu.edu.vn; 2 Faculty of Applied Sciences, Ton Duc Thang University, Ho Chi Minh City 700000, Vietnam; 3 Department of Chemistry, Tamkang University, New Taipei City 25137, Taiwan; peter831119@gmail.com; 4 Institute of Biotechnology and Environment, Tay Nguyen University, Buon Ma Thuot 630000, Vietnam; nguyenhanh2208.tn@gmail.com (T.H.N.); nadzungtaynguyenuni@yahoo.com.vn (A.D.N.); 5 Department of Science and Technology, Tay Nguyen University, Buon Ma Thuot 630000, Vietnam; nguyenminhtrung2389@gmail.com (M.T.N.); thanhthaotnu@gmail.com (T.T.T.T.); doanthng@gmail.com (C.T.D.); tranngoctnu@gmail.com (T.N.T.); 6 Division of Chinese Materia Medica Development, National Research Institute of Chinese Medicine, Taipei 11221, Taiwan; kuoyh@nricm.edu.tw; 7 Department of Chemical and Materials Engineering, Tamkang University, New Taipei City 25137, Taiwan

**Keywords:** α-Chitin, prodigiosin, anti-tumors, *Serratia marcescens*, bioprocessing

## Abstract

Marine chitins (MC) have been utilized for the production of vast array of bioactive products, including chitooligomers, chitinase, chitosanase, antioxidants, anti-NO, and antidiabetic compounds. The aim of this study is the bioprocessing of MC into a potent anticancer compound, prodigiosin (PG), via microbial fermentation. This bioactive compound was produced by *Serratia marcescens* TKU011 with the highest yield of 4.62 mg/mL at the optimal conditions of liquid medium with initial pH of 5.65–6.15 containing 1% α-chitin, 0.6% casein, 0.05% K_2_HPO_4_, and 0.1% CaSO_4_. Fermentation was kept at 25 °C for 2 d. Notably, α-chitin was newly investigated as the major potential material for PG production via fermentation; the salt CaSO_4_ was also found to play the key role in the enhancement of PG yield of *Serratia marcescens* fermentation for the first time. PG was qualified and identified based on specific UV, MALDI-TOF MS analysis. In the biological activity tests, purified PG demonstrated potent anticancer activities against A549, Hep G2, MCF-7, and WiDr with the IC_50_ values of 0.06, 0.04, 0.04, and 0.2 µg/mL, respectively. Mytomycin C, a commercial anti-cancer compound was also tested for comparison purpose, showing weaker activity with the IC_50_ values of 0.11, 0.1, 0.14, and 0.15 µg/mL, respectively. As such, purified PG displayed higher 2.75-fold, 1.67-fold, and 3.25-fold efficacy than Mytomycin C against MCF-7, A549, and Hep G2, respectively. The results suggest that marine chitins are valuable sources for production of prodigiosin, a potential candidate for cancer drugs.

## 1. Introduction

Chitin, an abundant material, has been widely produced from fishery processing byproducts. Of the natural chitin-containing materials, shrimp shells, squid pens, and crab shells have the highest chitin content [1], and as such, are used for chitin production. Chitin and its derivatives display great economic value thanks to their versatile activities and potential biotechnological applications, and chitin-containing materials have been reported to be used for the production of a vast array of bioactive products, such as exopolysaccharides [2,3,4], chitooligomers [5], antioxidants [6,7], biofertilizers [8] insecticidal materials [9,10], and biosorbents [11,12]. Recently, these chitin-containing materials were extensively used for the production of antidiabetic drugs [13,14,15,16,17,18]. In this study, chitinous materials were utilized for the production of prodigiosin, an active anticancer compound, via microbial fermentation. 

Prodigiosin (PG), a red pigment is a typical alkaloid constituent produced by several bacterial genus, *Serratia marcescens* and some other Gram-negative bacterial strains [19]. PGs have been recognized as bioactive bacterial metabolites with vast reported valuable bioactivities, including antibacterial, cytotoxic, antifungal, algicidal, antiprotozoal, antimalarial, antiproliferative, anticancer [19,20,21,22], antioxidant, and immunosuppressant [23] activities. PG also has been uniquely used as a natural based dye for textiles and olefins [24]. 

Due to the wild range of unique applications of PG, the production studies on this bioactive compound have been received with great interest [13,24], and many types and selective media have been investigated for PG production via microbial fermentation, such as a medium containing 2% sodium oleate [25], maltose broth, nutrient broth containing sesame seeds, peptone glycerol broth [26], nutrient broth, nutrient broth with 0.5% maltose or 0.5% glucose, powdered peanut seed broth [27], corn steep mannitol medium, mannitol medium, corn steep medium, Cassava waste mannitol medium, cassava waste medium, and luria bertani glucose medium [23]. For low cost production of PG, we established the PG production from marine chitinous wastes [9,10,11]. In these studies, various marine chitinous materials, including crab shells, shrimp shells, shrimp heads and squid pens were used as the sole carbon/nitrogen source; squid pens were found to be the most effective material for PG production by *S. marcescens*. However, numerous scientific parameters were not investigated in our previous studies, such as the kind of marine chitin (α or β), protein sources, chitin/protein ratio, and supplementary minerals for the best PG productivity production by *S. marcescens.* All those previously unknown items were newly investigated in this study, and the PG produce from the medium containing marine chitin was also evaluated for its effect on four cancerous cell lines—A549, Hep G2, MCF-7, and WIDR—in this report.

## 2. Results and Discussion

### 2.1. New Records of Marine α-Chitin as the Potential Carbon Source for Prodigiosin Synthesis by Serratia marcescens

**Figure 1:**
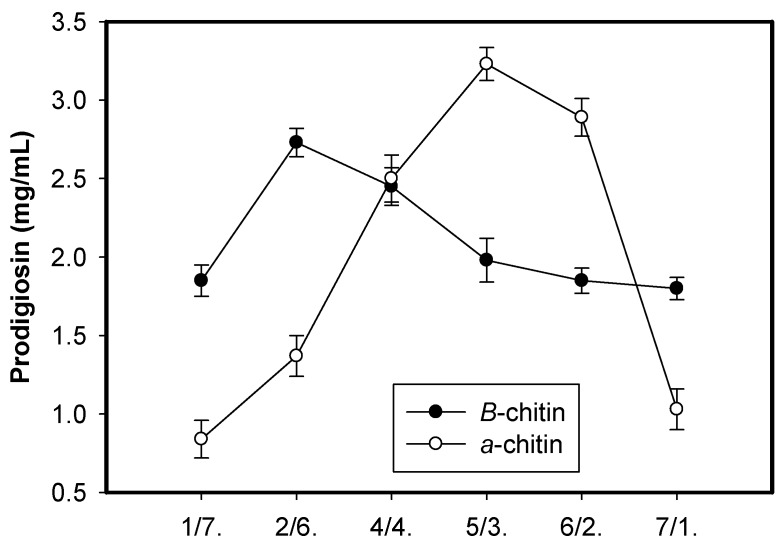
The effect of chitin/protein ratio. The chitin including two forms of α-chitin and β-chitin were mixed with free protein (casein) with six ratios of 1/7, 2/6, 4/4, 5/3, 6/2, and 7/1 (*w/w*) and used as the sole carbon and nitrogen source with the concentration of 1.5% (*w/v*). The fermentation was performed at the conditions at 30 °C in 1 d, and then at 25 °C in next 2 d, shaking speed of 150 rpm, and a ratio volume of medium: flask of 1:2.5 (*v/v*).

Carbon source has been proven to play an important role in PG production via microbial fermentation [19]. In previous studies, squid pens powder (SPP) was found to be cost-effective material for the production of PG by *Serratia marcescens* TKU011, compared to other materials; SPP was reported to contain approximately 60% chitin and 40% protein [13]. Thus, chitin contained in SPP may prove a significant factor in PG production. To investigate the role of chitin as well as its combination with free protein in different ratios on the PG production by *S. marcescens*, the chitins obtained from SPP (β-chitin) and shrimp shells (α-chitin) by using the method reported by Wang et al., 2006 [28] were mixed with free protein (casein) with the ratio (chitin/casein) of 7/1, 6/2, 5/3, 4/4, 2/6, and 1/7 (*w/w*) and used as the sole carbon and nitrogen source for fermentation by *S. marcescens* TKU011; SPP was also used as the control for comparison purpose. The results in Figure 1 show that β-chitin mixed with casein with the ratio of 2/6 (*w/w*) give higher PG yield production (2.73 mg/mL) than that of SPP (2.45 mg/mL) fermented by *S. marcescens* TKU011, while α-chitin mixed with free protein at the ratio of 5/3 (*w/w*) reach the greatest PG yield production of 3.23 mg/mL. In addition to the use of α-chitin providing higher PG yield, and α-chitin could be more abundantly obtained from vast resources (crab shells, shrimp shells, etc.) than β-chitin (mainly obtained from squid pens); thus, α-chitin was chosen for our further investigation. Based on the recent literature review, PG has been produced by *S. marcescens* with various types of carbon/nitrogen sources [9,10,11,13,14,25,26,27,28,29,30,31,32,33]; however, very few studies report on the use of chitinous materials (squid pens) [19] as the carbon/nitrogen source for PG production; for the first time in this study, α-chitin obtained from marine resources were investigated as a potent carbon source for high scale PG production via microbial fermentation.

For the comparison of the PG producing by different *S. marcescens* strains, a total four strains including *S. marcescens* TKU011, *S. marcescens* CC17, *S. marcescens* TNU01, and *S. marcescens* TNU02 were conducted for fermentation. As shown in the Table 1, *S. marcescens* TKU01, *S. marcescens* TNU01, and *S. marcescens* TNU02 showed their same level in PG production with the PG yield of 325–335 mg/100mL, and 236–243 mg/100mL when the medium contained newly designed C/N source (0.94% α-chitin and 0.56% Casein), and 1.5% squid pens, respectively. *S. marcescens* CC17 demonstrated the lowest production of PG yield.

To date, PG has been produced from many carbon sources with multiple designed media [9,10,11,13,14,25,26,27,28,29,30,31,32,33]. As summarized in Table 2, the designed medium gave the PG productivity in the scale of 0 up to around 200 (mg/100mL). With the medium containing 0.94% α-chitin + 0.56% Casein, PG produced by *S. marcescens* TKU011 strain reached 335 (mg/100mL). However, two previous studies reported that with 2.0% sesame seed [26] and the medium containing 6.97 g/L of peanut powder, 11.29 mL/L of olive oil and 16.02 g/L of beef extract [33] even reached the PG yield of 1668 and 1362.2 mg/100mL, respectively. These are cases with 4.979-fold and 4.066-fold higher yield than PG yield produced by *S. marcescens* TKU011 in this study. In these above cited reports [26,33], the PG-producing bacteria may be unique strains and the qualification of PG in these studies were not described in detail. 

### 2.2. Optimization of Culture Conditions for Enhancement of Prodigiosin Production by Serratia marcescens

**Figure 2:**
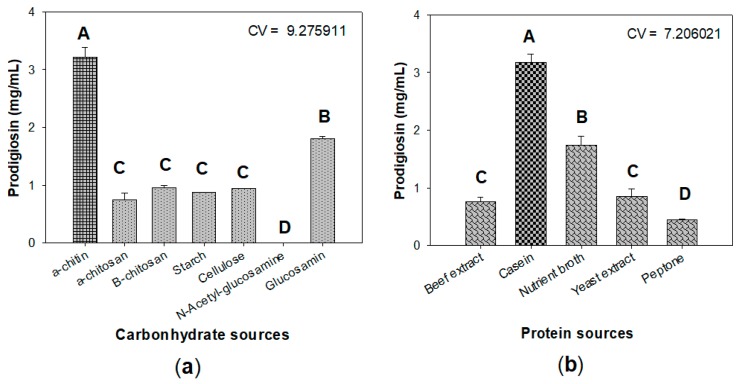
The effect of carbohydrate (**a**) and protein (**b**) sources on the prodigiosin production by *S. marcescens* TKU11. Carbohydrate and protein were mixed at the ratio of 5/3 and used as the C/N source. The culture medium contains 1.5% C/N source, 0.1% K_2_HPO_4_ and 0.1% FeSO_4_(NH_4_)_2_SO_4._ The fermentation was performed at the conditions at 30 °C in 1 d, and then at 25 °C over the next 2 d, shaking speed of 150 rpm, and a ratio volume of medium:flask of 1:2.5 (*v/v*). Means of prodigiosin yield (mg/100mL) values with the same letter in each figure are not significantly different based on Duncan’s multiple range test (alpha = 0.01).

To investigate the effect of C/N sources on PG production by *S. marcescens* TKU011, some chitinous materials, including chitosan (a derivative of chitin), *N*-acetyl-glucosamine (monomer of chitin), glucosamine (mono of chitosan), and some other carbon sources, such as cellulose and starch, were used for fermentation. As shown in Figure 2a, among various tested carbon sources, α-chitin displayed the most suitable substrate for PG production by *S. marcescens* with the greatest yield of 3.21 mg/mL, followed by its monomer *N*-acetyl-glucosamine with the PG yield production of 1.81 mg/mL, and all other tested carbon source give low yield PG production (≤ 0.96 mg/mL). Thus, α-chitin was chosen as an excellent substrate for further investigation. To further investigate the effect of the combination of α-chitin and free protein source, a total of five protein sources—beef extract, casein, nutrient broth, yeast extract, and peptone—were combined with α-chitin used as sole C/N source for fermentation by *Serratia marcescens* to produce PG. The experimental results in Figure 2b showed that the combination of casein and α-chitin gave a significantly higher yield of PG (3.31 ± 0.142 mg/mL) than other combinations (≤ 1.73 ± 0.166 mg/mL); casein was chosen for combination with α-chitin and used as the sole C/N source in our nest experiments. Casein was also found to be a suitable nitrogen source for producing PG with high level yield in several previous studies, such as maltose/casein and sucrose/casein with ratio 1/1 leading to PG yield production of 2.354 and 3.12 mg/ml, respectively [34]; 2% oral casein was mixed with some salts and used as the medium for fermentation to produce a high yield of PG with 4.28 mg/mL [23]. Differing from previous studies, we established the novel medium with the combination of abundant chitinous material (α-chitin) and casein with the ratio of 5/3 (*w/w*). This designed medium also reached the high PGs yield of 3.21 mg/mL, and as such used for next investigation. 

**Figure 3:**
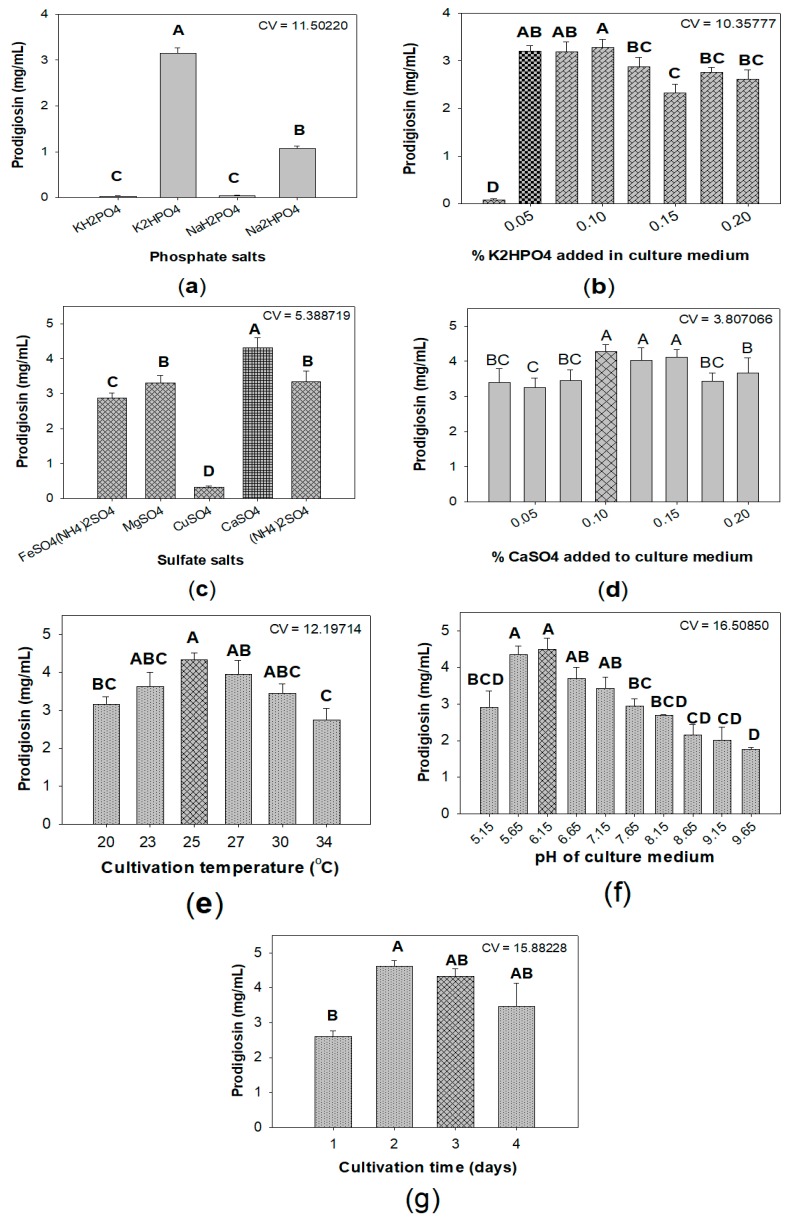
The effect of phosphate salts (**a**,**b**), sulfate salts (**c**,**d**), cultivation temperature (**e**), pH of culture medium (**f**), and cultivation time (**g**) on the prodigiosin production by *S. marcescens* TKU011. Medium with the combination of α-chitin and casein with the ratio of 5/3 (*w/w*). The culture medium containing 1.5% C/N source, 0.1% FeSO_4_(NH_4_)_2_SO_4_ (**a**,**b**), 0.05% K_2_HPO_4_ (**c**,**d**). The fermentation was performed at 30 °C in 1 d, and then at 25 °C in the next 2 d, shaking speed of 150 rpm, and a ratio volume of medium: flask of 1:2.5 (*v/v*). Means of prodigiosin yield (mg/100mL) values with the same letter in each figure are not significantly different based on Duncan’s multiple range test (alpha = 0.01).

Some previous studies indicated that salt ingredients in medium, especially phosphate and sulfate salts, played a vital role in enhancing the yield of PG [9,19,35]. As shown in Figure 3a, among various tested salts, K_2_HPO_4_ was found to be the most suitable phosphate salt for PG biosynthesis by *S. marcescens.* Further experiments investigated the optimal added K_2_HPO_4_ was 0.05% (Figure 3b). This result is in agreement with the previous reports [19,36]. This added concentration of K_2_HPO_4_ was used to mix with various kinds of sulfate salts, including FeSO_4_(NH_4_)_2_SO_4_, MgSO_4_, CaSO_4_, CuSO_4_, (NH_4_)_2_SO_4_ as the basal salt solution. CaSO_4_ demonstrated good effect on PG production with the highest yield of 4.32 mg/mL (Figure 3c). The final experiment (Figure 3d) found that CaSO_4_ added at its of 0.1% to medium is the optimal concentration. Notably, CaSO_4_ was newly investigated as potent salt added to significantly enhance PG production by *S. marcescens.*

To achieve maximum production of prodigiosin, some parameters, including cultivation temperature (Figure 3e), initial pH of medium (Figure 3f), and period of cultivation time (Figure 3g), were also investigated for their effect on PG yield produced by *S. marcescens.* Overall, *S. marcescens* TKU011 produce highest PG (4.62 mg/mL) in liquid medium with initial pH of 5.65–6.15 containing 1% α-chitin, 0.6% casein, 0.05% K_2_HPO_4_, and 0.1% CaSO_4_, fermentation was kept at 25 °C for 2 d. 

### 2.3. Purification and Qualification of Prodigiosin from Fermented Medium Containing α-Chitin

**Figure 4:**
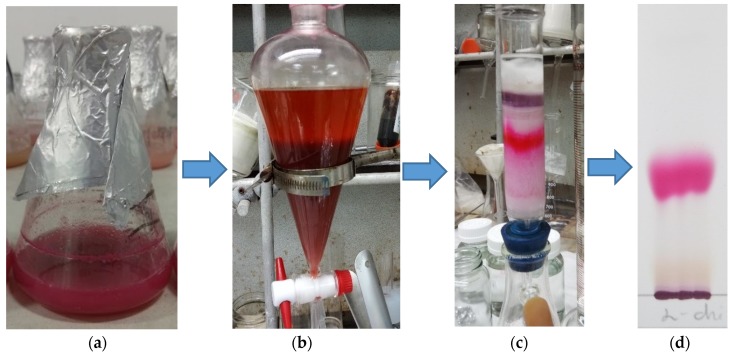
The purification and isolation process of prodigiosin. The culture broth containing prodigiosin (**a**) was extract by ethyl acetate (**b**). The crude prodigiosin was further separated by silica gel column (**c**) and then isolated by thin layer chromatography (TLC) (**d**).

α-chitin was mixed with casein with the ratio of 5/3 (*w/w*) and used as the sole C/N source at the concentration of 1.5% (*w/v*) for fermentation by *S. marcescens* TKU011. PG was primary extracted from the cultured broth by ethyl acetate. The PG from the cell pellet extracted with acetone was mixed with the ethyl acetate layer. After evaporation to dry crude PG, this compound was further purified via silica gel column, and then finally isolated by thin layer chromatography. The procedure is summarized in Figure 4.

**Figure 5:**
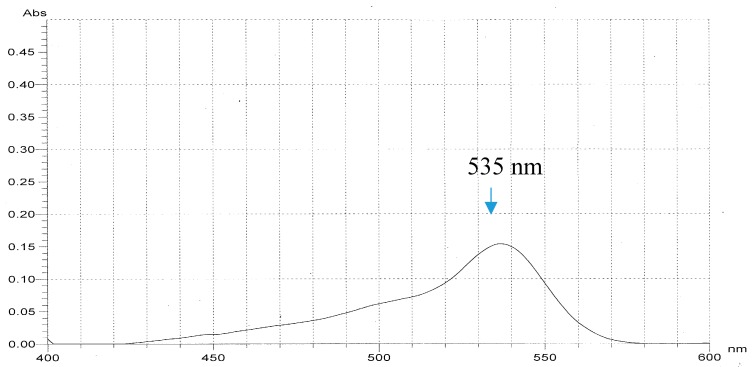
UV spectrum of purified prodigiosin newly biosynthesized from the novel designed substrate (α-chitin/casein = 5/3) by *S. marcescens* TKU011.

**Figure 6:**
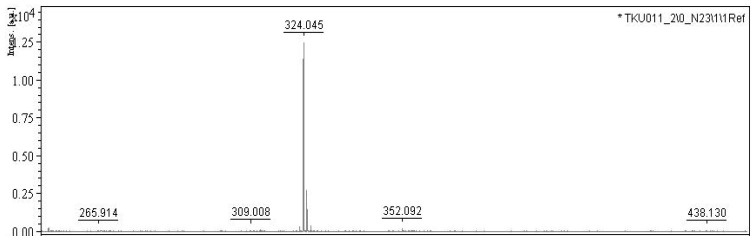
MALDI-TOF MS (Matrix-Assisted Laser Desorption Ionization - Time of Flight Mass Spectrometry) spectra of purified prodigiosin. 2,5-dihydroxybenzoic acid was used as a matrix in CAN-TFA-H_2_O solution (50/0.1/50%, *v/v/v*) to separate the sample in the MALDI-TOF instrument (Bruker Daltonics, Bremen, Germany) with a nitrogen laser emitting at 337 nm, operating in linear mode. Each spectrum of mass was calculated based on the data of around 30–50 laser shots, and external calibration with three points was used for assignment of mass [36].

In our previous report [9], the *S. marcescens* TKU011 prodigiosin was identified via its UV absorption, molecular, and ^1^H-NMR spectrum. Due to the prodigiosin produced by the same strain, in this study, we reconfirm this purified compound by some rapid method including UV spectra and MALDI-TOF MS analysis. The purified compound demonstrated significant absorption spectroscopy at 535 nm (Figure 5) and the MALDI-TOF MS revealed a molecular weight of 324 Da for the purified PG (Figure 6), which are the specific absorption weight length and molecular weight of prodigiosin [9,33]. 

### 2.4. Evaluation of Inhibitory Effect of Prodigiosin against Cancerous Cell Lines Models

Prodigiosin has been investigated for its vast biological activities, including insecticidal, antioxidant, algicidal, antimicrobial, anti-inflammatory, antimalarial, anticancer, immunomodulatory, and anti-diabetic activities [19,37]. With the aim of evaluating the prodigiosin newly synthesized in this study for potential use in anticancer drugs, prodigiosin was tested for its inhibition against some cancerous cell lines, including A549, Hep G2, MCF-7, and WiDr. As presented in Table 3, prodigiosin produced from the novel medium with the combination of α-chitin and casein used as the C/N source demonstrated its highly effective inhibition against all tested cancerous cell lines with great inhibition values (%) in the range of 90.2–93.9% at the tested concentration at 10 µg/mL. These anticancer inhibition values of prodigiosin were comparable to those of Mitomycin C, a commercial anticancer compound (91.7–94.1%). The crude sample also showed potent activity with max inhibition values in the range of 79.4–93.2% at the concentration of at 10 µg/mL. 

The samples were tested at their concentration of 10 µg/mL for their anticancer activity against MCF-7 (Human breast adenocarcinoma), A549 (Human lung carcinoma), Hep G2 (Human hepatocellular carcinoma), and WiDr (Human colon adenocarcinoma). The means of inhibition (%) with the same letter are not significantly different based on Duncan’s multiple range test (alpha = 0.01). CV (%) = 1.979533.

To clarify the potential effect of prodigiosin against these tested cancerous cells, the samples were diluted and tested in various concentrations; the result of anticancer activity was then calculated and presented as IC_50_ value. IC_50_ value is a concentration of sample that may reduce 50% of cancerous cells; therefore, the smallest this value of the sample, the strongest anticancer activity it displayed. As shown in Table 3, prodigiosin strongly inhibited all 4 tested cancerous cells with very low IC_50_ values of 0.04, 0.06, 0.04, and 0.20 against A549, Hep G2, MCF-7, and WiDr, respectively (Table 4). The anticancer activity of the purified prodigiosin is clearly higher than that of the crude sample with IC_50_ values in the range of 0.38–0.88 µg/mL. Mitomycin C was also tested in comparison and showed its inhibition against A549, Hep G2, MCF-7 and WiDr with IC50 values of 0.11, 0.10, 0.13, and 0.10 µg/mL, respectively. In comparison, prodigiosin displayed significantly higher inhibition against A549, Hep G2, and MCF-7 but weaker inhibition against WiDr than Mitomycin C. 

To date, various prodigiosin compounds produced via fermentation reported anticancer activity against MCF-7 and A549. Prodigiosin was also reported showing inhibition against Hep G2 in several reports [38,39]. However, no available data report the potent inhibition of prodigiosin against human colon adenocarcinoma WiDr cell line. Specifically, the purified prodigiosin produced from marine chitin materials in the current study demonstrated high level anticancer activity. It displayed higher 2.75-, 1.67-, and 3.25-fold efficacy than commercial anticancer compounds against MCF-7, A549, and Hep G2, respectively. 

Anticancer drugs have been extensively investigated for years. Commercially available anticancer drugs obtained from chemical synthesis show strong activity but result in various side effects [40]. Thus, the investigation of natural anticancer drugs has received much interest. Prodigiosin was investigated as a potent natural anticancer agent since it showed strong inhibition against a wide range of human cancer cell lines but lower toxicity toward normal cells [41]. The mechanisms of anticancer activity of prodigiosin were reported in previous studies [41,42,43]. Prodigiosin induces apoptosis in various human cancer cells [42], and some possible mechanisms were prosed that prodigiosin as mitogen-activated protein kinase regulators, cell cycle inhibitors, DNA cleavage agents, and pH modulators [43]. The results in this study contributed to announce the novel anticancer activity of prodigiosin against WiDr cell line and also reconfirmed that prodigiosin as effective anticancer agents.

## 3. Materials and Methods

### 3.1. Materials

*Serratia marcescens* TKU011 was obtained from our previous study [9]. *S. marcescens* CC17 obtained from the previous study [44], *S. marcescens* TNU01 and *S. marcescens* TNU02 were newly isolated from the soils of Buon Ma Thuot City, Vietnam, and identified based on 16S gene sequence in this study. Shrimp shell and squid pens were purchased from Fwu-Sow Industry (Taichung, Taiwan). Four cancerous cell lines—MCF-7 (Human breast adenocarcinoma), A549 (Human lung carcinoma), Hep G2 (Human hepatocellular carcinoma), and WiDr (Human colon adenocarcinoma)—were purchased from the Bioresources Collection and Research Centre (Hsinchu, Taiwan). Mitomycin C and Silicagel (Geduran^®^ Si 60 for column chromatography, size: 0.040–0.063 mm) were obtained from Sigma Chemical Co. (St. Louis City, MO, USA) and Mitsubishi Chemical Co. (Tokyo, Japan), respectively. Reagents, solvents and common chemicals were used at the highest grade available.

16S gene sequence of *Serratia marcescens* TNU01: 

TGGCTCAGATTGAACGCTGGCGGCAGGCTTAACACATGCAAGTCGAGCGGTAGCACAGGGGAGCTTGCTCCCTGGGTGACGAGCGGCGGACGGGTGAGTAATGTCTGGGAAACTGCCTGATGGAGGGGGATAACTACTGGAAACGGTAGCTAATACCGCATAACGTCGCAAGACCAAAGAGGGGGACCTTCGGGCCTCTTGCCATCAGATGTGCCCAGATGGGATTAGCTAGTAGGTGGGGTAATGGCTCACCTAGGCGACGATCCCTAGCTGGTCTGAGAGGATGACCAGCCACACTGGAACTGAGACACGGTCCAGACTCCTACGGGAGGCAGCAGTGGGGAATATTGCACAATGGGCGCAAGCCTGATGCAGCCATGCCGCGTGTGTGAAGAAGGCCTTCGGGTTGTAAAGCACTTTCAGCGAGGAGGAAGGTGGTGAACTTAATACGTTCATCAATTGACGTTACTCGCAGAAGAAGCACCGGCTAACTCCGTGC.

16S gene sequence of *Serratia marcescens* TNU02: 

CTGGCTCAGATTGAACGCTGGCGGCAGGCTTAACACATGCAAGTCGAGCGGTAGCACAGGGGAGCTTGCTCCCCTGGGTGACGAGCGGCGGACGGGTGAGTAATGTCTGGGAAACTGCCTGATGGAGGGGGATAACTACTGGAAACGGTAGCTAATACCGCATAACGTCGCAAGACCAAAGAGGGGGACCTTCGGGCCTCTTGCCATCAGATGTGCCCAGATGGGATTAGCTAGTAGGTGGGGTAATGGCTCACCTAGGCGACGATCCCTAGCTGGTCTGAGAGGATGACCAGCCACACTGGAACTGAGACACGGTCCAGACTCCTACGGGAGGCAGCAGTGGGGAATATTGCACAATGGGCGCAAGCCTGATGCAGCCATGCCGCGTGTGTGAAGAAGGCCTTCGGGTTGTAAAGCACTTTCAGCGAGGAGGAAGGTGGTGAACTTAATACGTTCATCAATTGACGTTACTCGCAGAAGAAGCACCGGCTAACTCCGTG C.

### 3.2. Fermentation for Prodigiosin Biosynthesis by S. marcescens TKU011

α-chitin and β-chitin obtained from shrimp shells and squid pens were mixed with casein with 6 ratios of 1/7, 2/6, 4/4, 5/3, 6/2, and 7/1 (*w/w*) and used as the sole carbon and nitrogen source with the concentration of 1.5% (*w/v*) for fermentation. The medium containing 1.5% carbon and nitrogen source, 0.1% K_2_HPO_4_ and 0.1% FeSO_4_(NH_4_)_2_SO_4_ was fermented by *S. marcescens* TKU011 at 30 °C in 1 d, and then 25 °C over the next 2 d, shaking speed of 150 rpm, and a ratio volume of medium:flask of 1:2.5 (*v/v*). α-chitin mixed with casein at the ratio of 5/3 (*w/w*) reached the greatest PG yield production; as such, α-chitin/casein was used for comparison in the following experiments evaluating other carbohydrate sources (chitosan, *N*-acetyl-glucosamine, glucosamine, cellulose and starch) and proteinous sources. α-chitin/casein at the ratio of 5/3 (*w/w*) finally proved best and was used for fermentation in the subsequent investigation, including the effect of added salts and some parameters.

The effect of phosphate salts and its optimal concentration added to culture medium on PG production: four kinds of phosphate salts—KH_2_PO_4_, K_2_HPO_4_, NaH_2_PO_4_, and Na_2_HPO_4_—were used. The medium containing 0.1% FeSO_4_(NH_4_)_2_SO_4_, 0.1% phosphate salt, and 1.5% C/N source were fermented at 30 °C in 1 d, and then at 25 °C over the next 2 d, shaking speed of 150 rpm, and a ratio volume of medium:flask of 1:2.5 (*v/v*). KH_2_PO_4_ was found to be the most suitable phosphate salt; as such, it was used to investigate its optimal concentration added to the medium. 0.025, 0.05, 0.1, 0.125, 0.15, 0.175, and 0.2% K_2_HPO_4_ and combined with 0.1% FeSO_4_(NH_4_)_2_SO_4_; the fermentation procedure was conducted at 30 °C in 1 d, and then 25 °C over the next 2 d, shaking speed of 150 rpm, and a ratio volume of medium:flask of 1:2.5 (*v/v*).

The effect of sulfate salts and its optimal concentration added to culture medium on PG production: FeSO_4_(NH_4_)_2_SO_4_, MgSO_4_, CaSO_4_, CuSO_4_, and (NH_4_)_2_SO_4_ were used as sulfate salts. The medium containing 0.05% K_2_HPO_4_ and 0.1% sulfate salt, 1.5% C/N source were fermented at 30 °C in 1 d, and then 25 °C over the next 2 d, shaking speed of 150 rpm, and a ratio volume of medium:flask of 1:2.5 (*v/v*). CaSO_4_ was found to be the most suitable sulfate salt; as such, it was used to investigate its optimal concentration added to the medium. 0.025, 0.05, 0.1, 0.125, 0.15, 0.175, and 0.2% CaSO_4_ and combined with 0.1% K_2_HPO_4_; the fermentation was conducted at 30 °C in 1 d, and then 25 °C over the next 2 d, shaking speed of 150 rpm, and a ratio volume of medium:flask of 1:2.5 (*v/v*).

The effect of some parameters on PG production: some parameters including temperature programs (activated at 20, 23, 25, 27, 30, and 34 °C, and then fermented at 25 °C over the next 2 d), initial pH (5.15, 5.65, 6.15, 6.65, 7.15, 7.65, 8.15, 8.65, 9.15, 9.65) and period of cultivation time (0, 1, 2, 3, and 4 d). 

### 3.3. Prodigiosin Quilification and Purification

PG concentration was determined according to the method previously described by Wang et al., 2012 [36]. A mixture including 0.5 mL of fermented medium broth and 4 mL of methanol was vortexed. 2% (*w/v*) hydrated potassium aluminum sulfate was added into this mixture, mixed, and then centrifuged at 1400 g for 5 min. The harvested supernatant was then mixed with a solution of methanol/0.5 N HCl at the ratio of 1/9, *v/v*. The final solution optical density was measured at 535 nm. PG purified from the culture broth was used as the standard to convert OD535 nm measurement to mass concentration via an appropriate calibration. PG was purified by the method previously described [36] with modification. The culture broth was centrifugated at 10000× *g* for 15 min. The supernatant was collected and mixed with ethyl acetate with the ratio 1/1. The mixture was kept in a funnel for 3 h and immediately shaken every 30 min. The PG dissolved in ethyl acetate layer was collected. The PG from the cell pellet was extracted with acetone, and centrifugated at 10000× *g* for 15 min. The ethyl acetate layer and acetone containing PG were mixed, concentrated by evaporation of the solvent and then dissolved in ethyl acetate for further air oven drying at 55 °C to get dry crude PG powder. The crude PG was further purified by loading onto a silica open column (Geduran^®^ Si 60 (Merck KGaA, Darmstadt, Germany) for column chromatography, size: 0.040–0.063 mm) and eluted with methanol in chloroform with a ratio of 0/10–2/8 (*v/v*). The PG was finally isolated by thin layer chromatography (TLC) with the mobile phase system using methanol in chloroform with a ratio of 2/8 (*v/v*). After TLC separation, the lane contained PG was cut into small pieces, and methanol was used to dissolve PG. Then PG was concentrated in a rotary evaporator (IKA, Staufen, Germany) at 60 °C under vacuum. Finally, all the residue solvent was removed by keeping the sample in the oil pump in 12 h at 60 °C. The isolated PG was used to detect UV, MALDI-TOF MS and biological activities. 

### 3.4. Biological Activity Assays

Four cancerous cell lines: A549, Hep G2, MCF-7, and WIDR were conducted to evaluate the anticancer activities. The bioassay was done according to the methods described in detail in our previous report [6]. The significant differences of anticancer activity, including inhibition (%) and IC_50_ values, were analyzed with the use of Statistical Analysis Software (SAS-9.4) provided by the SAS Institute Taiwan Ltd. (Taipei City, Taiwan).

## 4. Conclusions

The current study established the novel designed medium containing 1% α-chitin, 0.6% casein, 0.05% K_2_HPO_4_, and 0.1% CaSO_4_ for efficient biosynthesis of bioactive prodigiosin. The fermentation was maintained at 25 °C for 2 d. The prodigiosin was purified, qualified via UV and Mass. The purified prodigiosin was also evaluated for its anticancer properties. Notably, the purified PG displayed high inhibition on four cancerous cell lines. The results in this study suggest that the purified prodigiosin newly biosynthesized may be a potential candidate for cancer drugs.

## Figures and Tables

**Table 1:** Comparison of the prodigiosin yield (mg/100mL) produced by different *Serratia marcescens* strains.

PG – Producing Strains	C/N Source	
	0.94% α-Chitin + 0.56% Casein	1.5% Squid Pens
*S. marcescens* TKU011	335 ± 14.4 ^a^	243 ± 24.8 ^b^
*S. marcescens* CC17	227 ± 2.93 ^b^	150 ± 5.77 ^c^
*S. marcescens* TNU01	329 ± 16.7 ^a^	240 ± 17.3 ^b^
*S. marcescens* TNU02	325 ± 14.4 ^a^	236 ± 20.8 ^b^
No bacteria	-	-

Means of prodigiosin yield (mg/100mL) values with the same letter are not significantly different based on Duncan’s multiple range test (alpha = 0.01). CV% = 4.271394. (-): no prodigiosin was detected.

**Table 2:** Comparison of the prodigiosin yield produced by *S. marcescens* in different reports.

PG – Producing Strains	C/N Source	Prodigiosin (mg/100mL)	Reference
*S. marcescens* TKU011	0.94% α-chitin + 0.56% Casein	335	This study
*S. marcescens* TKU011	1.5% squid pens	97.8	[9]
*S. marcescens* TKU011	1.5% peanut powder	116.8	[9]
*S. marcescens* TKU011	1.0% shrimp shells powders	19	[9]
*S. marcescens* TKU011	1.0% crab shells powders	11	[9]
*S. marcescens* TKU011	1.0% shrimp heads powders	3	[9]
*S. marcescens* TKU011	1.5% squid pens	248	[28]
*S. marcescens*	2.0% peanut seed	387.5	[26]
*S. marcescens*	2.0% peanut oil	289	[26]
*S. marcescens*	2.0% sesame seed	1668	[26]
*S. marcescens*	2.0% sesame oil	100.6	[26]
*S. marcescens*	2.0% copra seed	194	[26]
*S. marcescens*	2.0% coconut oil	142	[26]
*S. marcescens* SMΔR	Modified Luria-Bertani broth, 6.0% sunflower oil	79	[29]
*S. marcescens* SS-1	5 g/L yeast extract as sole N/C source	69	[30]
*S. marcescens* Nima	2% tryptone/glycerol (1/1)	12.5	[31]
*S. marcescens* Nima	100 mM 3-[*N*-morpholino]-ethanesulphonic acid	47.5	[32]
*S. marcescens* FZSF02	6.97 g/L of peanut powder, 11.29 mL/L of olive oil and 16.02 g/L of beef extract	1362.2	[33]
*S. marcescens* FZSF02	1% Soya peptone	117.4	[33]
*S. marcescens* FZSF02	1% Tryptone	35.3	[33]
*S. marcescens* FZSF02	1% Yeast extract	38.02	[33]
*S. marcescens* FZSF02	1% Fish meal	0	[33]
*S. marcescens* FZSF02	1% Soybean powder	0	[33]
*S. marcescens* FZSF02	1% Corn steep liquor	0	[33]

**Table 3:** Max inhibition against cancerous cell lines of prodigiosin.

	Max Inhibition Against Cancerous Cell Lines (%)			
	MCF-7	A549	Hep G2	WiDr
Crude sample	91.6 ± 1.76 ^a,b^	89.2 ± 1.43 ^b^	93.2 ± 2.12 ^a,b^	79.4 ± 1.72 ^c^
Purified Prodigiosin	92.5 ± 1.4 ^a,b^	92.6 ± 1.9 ^a,b^	93.9 ± 2.0 ^a^	90.2 ± 1.12 ^a,b^
Mitomycin C	94.1 ± 1.61^a^	93.3 ± 1.54 ^a,b^	91.7 ± 1.01 ^a^^,b^	92.6 ± 1.02 ^a,b^

The samples were tested at their concentration of 10 µg/mL for their anticancer activity against MCF-7 (Human breast adenocarcinoma), A549 (Human lung carcinoma), Hep G2 (Human hepatocellular carcinoma), and WiDr (Human colon adenocarcinoma). The means of inhibition (%) with the same letter are not significantly different based on Duncan’s multiple range test (alpha = 0.01). CV (%) = 1.979533.

**Table 4:** Anticancer activities of prodigiosin.

	Inhibition Against Cancerous Cell Lines (IC50, µg/mL)			
	MCF-7	A549	Hep G2	WiDr
Crude sample	0.44 ± 0.09 ^c,b^	0.46 ± 0.01 ^b^	0.38 ± 0.01 ^c,b^	0.88 ± 0.05 ^a^
Purified Prodigiosin	0.04 ± 0.01 ^e^	0.06 ± 0.01 ^e^	0.04 ± 0.01 ^e^	0.20 ± 0.03 ^d^
Mitomycin C	0.11 ± 0.01 ^e^	0.10 ± 0.01 ^e^	0.13 ± 0.01 ^e^	0.10 ± 0.01 ^e^

The samples were tested at their concentration range of 0.01–10 µg/mL for their anticancer activity against MCF-7 (Human breast adenocarcinoma), A549 (Human lung carcinoma), Hep G2 (Human hepatocellular carcinoma), and WiDr (Human colon adenocarcinoma). Means of IC_50_ (µg/mL) values with the same letter are not significantly different based on Duncan’s multiple range test (alpha = 0.01). CV% = 12.9069.
